# Effects of body mass index and range of motion on intraoperative change in pelvic tilt during total hip arthroplasty using the direct anterior approach

**DOI:** 10.1186/s12891-021-04087-x

**Published:** 2021-03-02

**Authors:** Masanori Okamoto, Masashi Kawasaki, Toshiaki Okura, Taisuke Seki, Shiro Imagama

**Affiliations:** 1 Department of Orthopaedic Surgery, Nagoya University Graduate School of Medicine, 65 Tsurumai-cho, Showa-ku, Nagoya, Aichi 466-8550 Japan; 2 Department of Orthopaedic Surgery, Aichi Koseiren Konan Kosei Hospital, Konan, Aichi Japan

**Keywords:** Total hip Arthroplasty, Direct anterior approach, Pelvic tilt, Accelerometer-based navigation system

## Abstract

**Background:**

Intraoperative pelvic tilt changes that occurs during total hip arthroplasty (THA) in the supine position affects cup placement and sometimes causes malalignment. The relationship between body mass index (BMI) and pelvic movement has been reported for some procedures, but not the direct anterior approach (DAA). The purpose of this study was to investigate intraoperative pelvic tilt changes that occurs during DAA.

**Methods:**

In this single-center, retrospective study, we reviewed 200 hips that underwent primary THA via DAA in the supine position using an accelerometer-based navigation system. Intraoperative changes in pelvic tilt and axial rotation from the start of surgery to cup placement were assessed using the navigation system. Preoperative clinical factors that increased pelvic tilt and axial rotation toward the surgical side by > 10° were analyzed via univariate and multiple logistic regression analyses.

**Results:**

The mean pelvic tilt value increased by 7.6° ± 3.8° (95% confidence interval [CI], 7.1–8.2; range, − 5.0–19.0) intraoperatively, and the axial rotation increased by 3.2° ± 2.7° (95% CI, 2.7–3.7; range, − 13.0–12.0). Univariate analysis revealed that the group with increased pelvic tilt showed significantly greater range of abduction and internal rotation, and significantly lower BMI than the group with no increased tilt. Pre-incisional pelvic tilt was significantly greater in the group with increased axial rotation than in the group with no increased rotation. On logistic regression analysis, BMI (odds ratio [OR], 0.889; 95% CI, 0.809–0.977; *p* = 0.014) and the range of internal rotation (OR, 1.310; 95% CI, 1.002–1.061; *p* = 0.038) were predictors of large increases in pelvic tilt. No predictors of large increases in axial rotation were identified.

**Conclusion:**

Significant forward pelvic tilt was observed in patients with a low BMI values and high ranges of internal rotation via THA using the DAA. Findings indicated that surgeons should pay attention to intraoperative pelvic movements, which may help identify patients with significant pelvic tilt changes.

## Introduction

Accurate cup positioning is important for achieving hip stability and good long-term outcomes in patients undergoing total hip arthroplasty (THA) [1, 2]. The position of the cup affects joint stability, polyethylene wear, ceramic damage, and risk of metallosis [3–5]. The concept of a “safe zone” for cup placement was reported by Lewnik in 1978 [6]. In recent years, some authors have reported that the ideal safe zone is narrower than that which has been traditionally reported [7, 8]. In addition, the range of anteversion is smaller than the range of inclination [9].

The direct anterior approach (DAA) is an intramuscular approach applied while the patient is positioned in the supine position. Advantages of DAA are as follows: minimal soft tissue damage and rapid postoperative recovery [10–12]. Cup positioning outside the safe zone may occur during THA via the DAA when the freehand technique without a navigation system is used [13]. Some reports show that cup anteversion is larger after using the DAA compared with the posterior approach [14, 15]. Pelvic movement during cup implantation is a major factor affecting cup positioning [16, 17]. The use of navigation systems for positioning of cups improves the accuracy of placement, reduces rates of dislocation, and minimizes the occurrence of aseptic revision of the acetabular component. Nevertheless, the proportion of THA procedures that use navigation is small, and the conventional procedure without navigation remains the gold standard [18]. Surgeons who perform conventional THA need to understand the significance of pelvic movements intraoperatively. It has been reported that in the supine position, pelvic tilt changes are associated with the patient’s body mass index (BMI) when using both lateral and anterolateral approaches [19, 20]. Interestingly, the relationship between BMI and pelvic tilt change of each approach differ. However, the associations between the DAA and BMI have not been reported.

HipAlign (OrthoAlign Inc., Aliso, U.S.A.) is an accelerometer-based, portable navigation system that is used for intraoperative pelvic tracking. The system displays the cup angle throughout THA in the supine position, and its use facilitates accurate cup placement [21]. On its display, the system indicates intraoperative pelvic motion in both sagittal and axial planes.

We hypothesized that the cup has a large anteversion angle that causes the pelvis to tilt forward (or lean) toward the operation side and that the functional pelvic plane at the start of surgery differs from the table plane during cup insertion. Thus, this study aimed to investigate intraoperative pelvic movements in THA via the DAA using an accelerometer-based navigation system to determine clinical factors associated with movement that results in abnormal cup positioning.

## Materials and methods

### Patients

The institutional review board approved this single-center, retrospective study. All study participants provided written informed consent. Primary THA for osteoarthritis was performed using the DAA on 256 consecutive hips of 235 patients from November 2017 to November 2019. Cementless or hybrid primary THA using the HipAlign portable navigation system was performed on 218 hips of 200 patients. Two hips with a history of osteotomy, 14 with simultaneous bilateral surgery, and 2 with data loss were excluded from the study; thus, we analyzed 200 hips of 189 patients. No patients experienced the loosening of pins that secured the HipAlign unit to the ilium.

### Surgical procedures

**Fig. 1:**
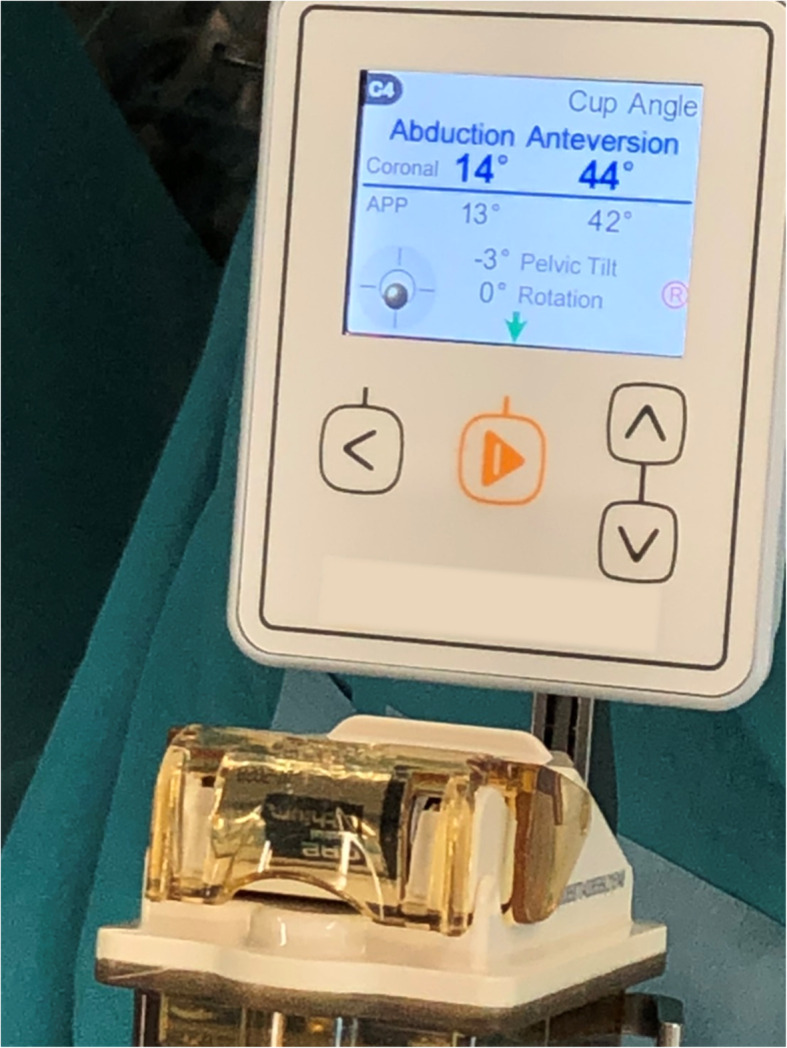
On the screen of the main unit, inclination and anteversion are displayed in radiographic values during cup insertion and after cup placement. Pelvic tilt and rotation are displayed at any given point in time

**Fig. 2:**
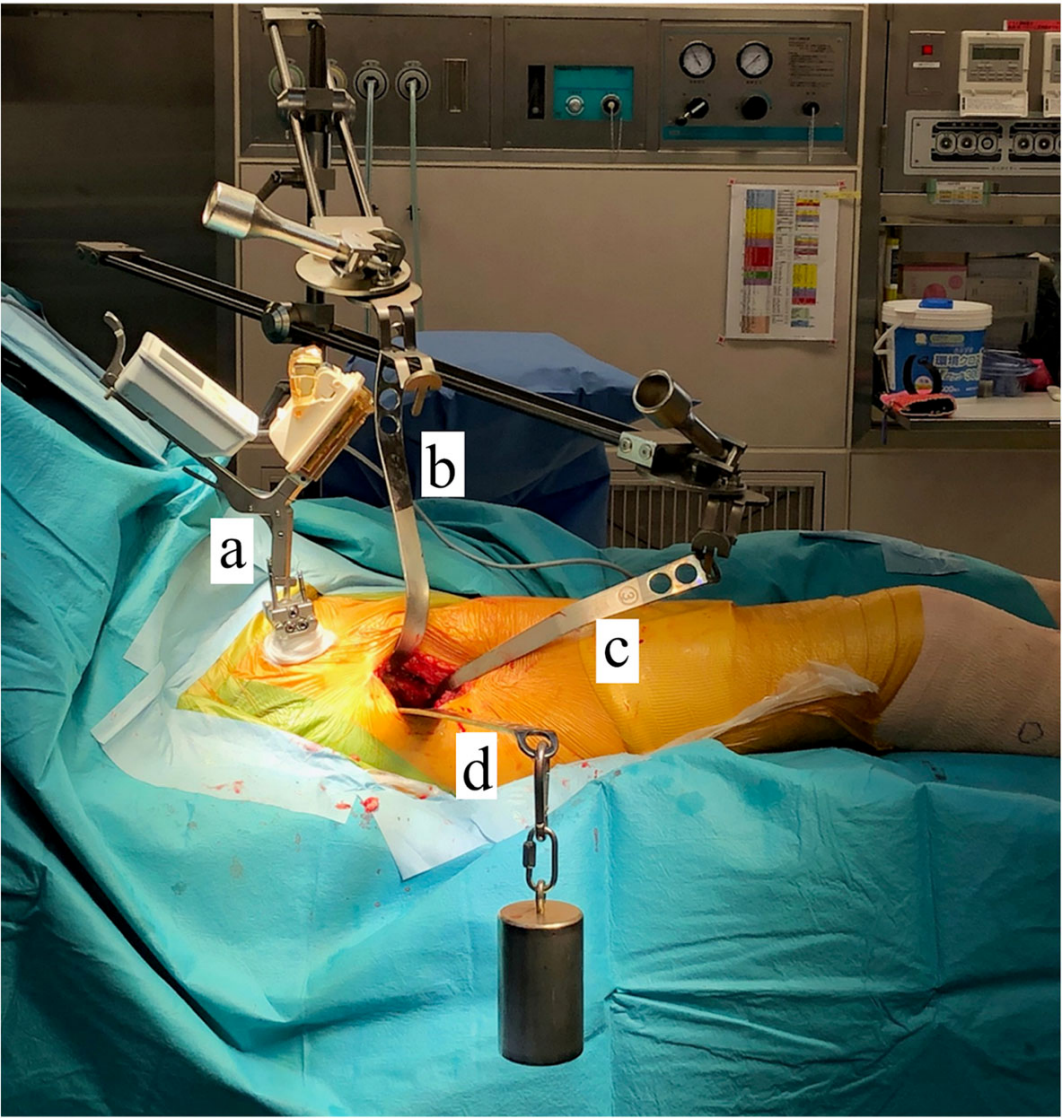
(**a**) HipAlign (OrthoAlign Inc., Aliso, U.S.A.) is placed on the iliac wing on the surgical side. In the picture, the sensor is attached to the unit. **b**, **c** The anterior retractor and distal retractor are held by a retractor holding device (Magic Tower, Zimmer-Biomet). **d** The posterior retractor is pulled using a 1.35 kg weight (DepuySynthes)

All surgeries were performed under general anesthesia, and the range of motion (ROM) of the hip in the supine position was measured before surgery. All THAs were performed via the same procedure by three surgeons using the DAA with the patient in the supine position on a standard operating table. HipAlign was used in accordance with a procedure previously described [21]. The main unit was placed on the iliac crest on the affected side, and registration was performed before the first skin incision. Two 4.0-mm fixation pins were inserted into the iliac crest on the affected side to fix the navigation unit. The direction of gravitational acceleration was then registered. The anterior superior iliac spine (ASIS) and pubic tubercle were registered as landmarks by placing the pointer on the overlying skin. Then, the sensor was attached to the main unit. Pelvic tilt and axial rotation with reference to the functional pelvic plane were displayed on the screen of main unit (Fig. 1). A 7-cm skin incision was made to enter the intermuscular plane between the tensor fasciae latae and sartorius muscles. Labrum resection, acetabular reaming, and cup placement were performed while preserving the lower portion of the anterior and posterior capsules with three acetabular retractors. The anterior and anterior-inferior retractors were pulled and held using Magic Tower (Zimmer Biomet Holdings. IN, USA), and the posterior retractor was pulled using a 1.35-kg weight (DepuySynthes, Warsaw, IN, USA) (Fig. 2). The capsule in the inferior band of the iliofemoral ligament was incised. The superior band of the iliofemoral ligament, ischiofemoral ligament, and the conjoined external rotator tendon were preserved.

**Fig. 3:**
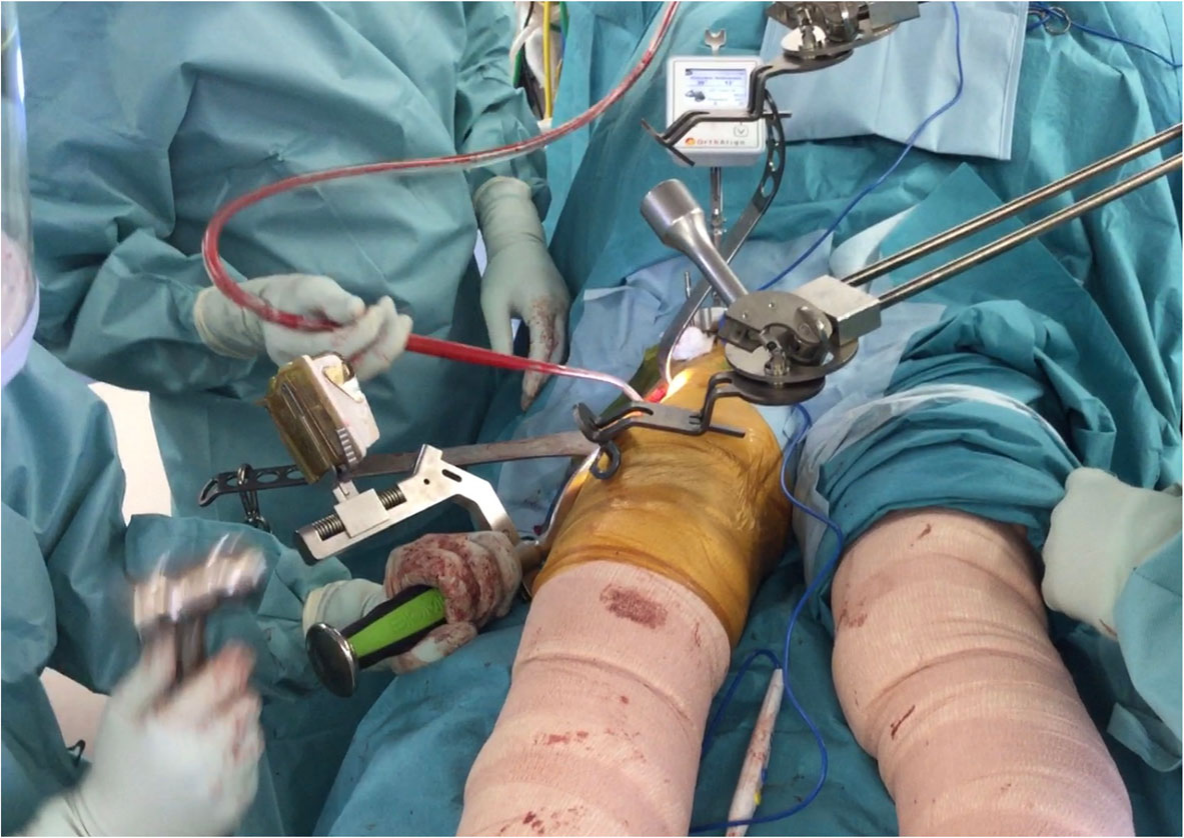
The sensor is fitted on the cup insertion handle. Inclination and anteversion are displayed in radiographic values on the screen of the main unit in real time

The cup was placed after determining the target angle, which was displayed on the screen of the navigation system. While looking at the display screen, the operator impacted the cup inserter with the hammer and placed the cup (Figs. 1, 3). The target angle of cup orientation was 40° inclination and 15° anteversion, which were based on radiographic definition. The following acetabular components and liners were used: G7 acetabular cup, and E1 or Biolox Delta ceramic liners (Zimmer Biomet Inc., Warsaw, IN, USA). The following femoral components were used: Taperloc Complete Microplasty, or CMK (Charnley Modified Kerboul) Original Concept stem (Zimmer Biomet Inc., Warsaw, IN, USA).

### Measurements

**Fig. 4:**
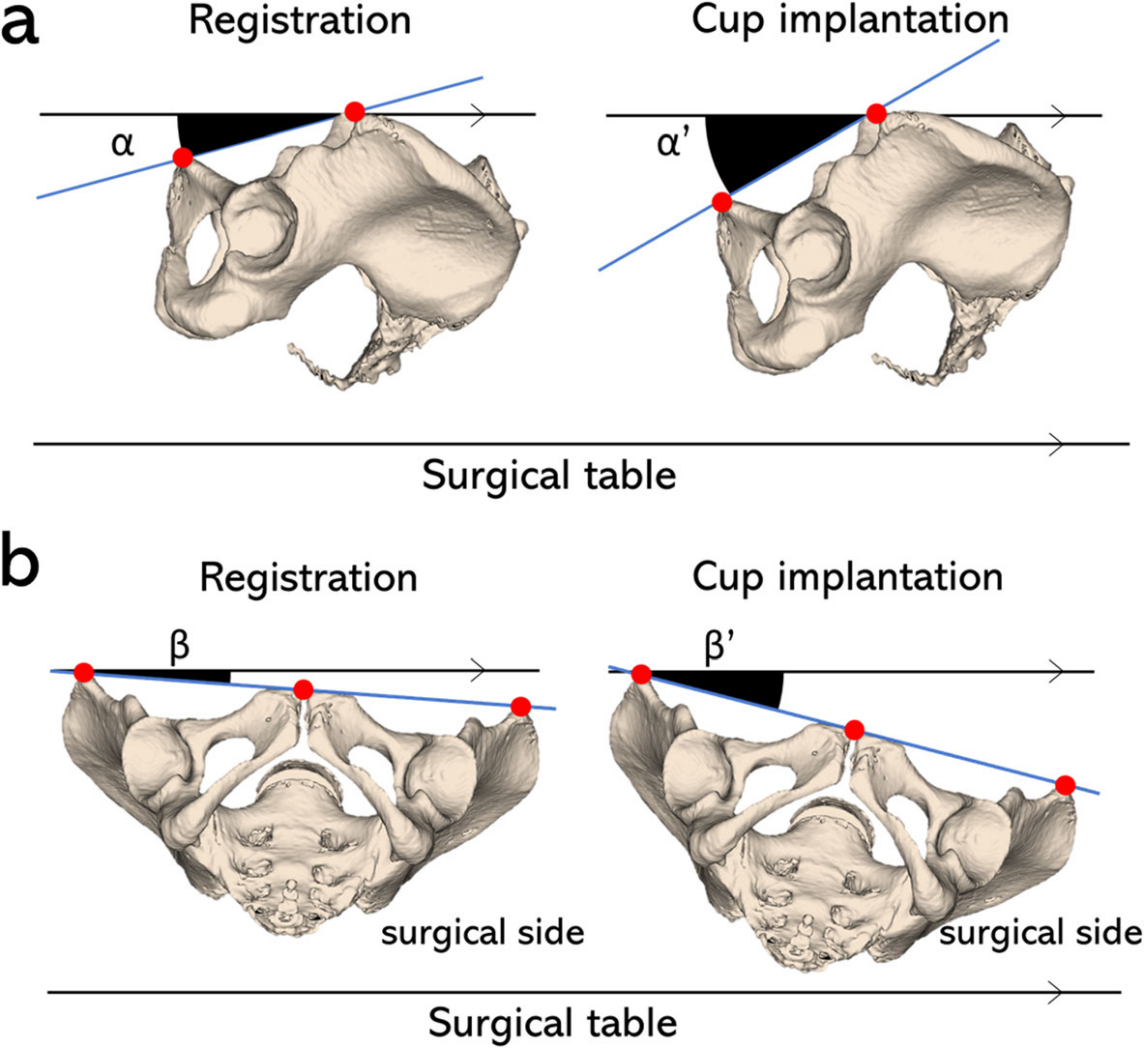
**a** Pelvic tilt is measured as the anterior tilt angle of the anterior pelvic plane in the sagittal plane toward the surgical table (α, α’). Pelvic tilt change is calculated by subtracting α from α’. Anterior tilt is given a positive value, and posterior tilt is negative. **b** Axial rotation is the lean of the anterior pelvic plane in the axal plane toward surgical side (β, β’). Lean toward the surgical side is given a positive value, and rise is negative. The difference in pelvic tilt toward the surgical side from registration to cup implantation is defined as change in axial rotation. The angle is calculated by subtracting β from β’

Pelvic angles were measured using the HipAlign system during navigation system registration at the time of cup placement to the acetabulum using the impactor, prior to removing the cup from the impactor. The angle of the sagittal plane against the horizontal plane was defined as the pelvic tilt. Forward tilt was positive, and posterior tilt was negative. The angle on the coronal plane was defined as axial rotation. The lean toward the surgical side was positive, and the rise was negative (Fig. 4). We measured changes in pelvic tilt and axial rotation from registration to cup placement. ROM was measured using a goniometer by two orthoepic surgeons (T.O and M.O). One person steadied the patient and the other stood on the evaluation side to measure relevant angles, which were recorded in degrees. For flexion measurements, only the leg to be measured was placed in maximum flexion. The evaluator placed one side of the goniometer horizontally on the surgical table and the other side parallel to the proximal thigh. Abduction and adduction were set to their maximum angles. The evaluator placed one side of the goniometer on the ASIS and measured the other side parallel to the thigh. For internal and external rotation, the patient’s hip was flexed 90°. When the angle of flexion was less than 90°, the patient was placed in maximum flexion. The angle between the lower leg axis and the body axis around the knee was measured. Extension was not measured, as the patient was under anesthesia in the supine position.

Two cohorts with pelvic movements were compared; those who presented with ≥10° change in pelvic tilt and axial rotation were placed into the increased tilt group, while those who presented with < 10° pelvic tilt and axial rotation were placed into the minor tilt group. The inclination or anteversion of the historical Lewnick’s safe zone of the cup was in the range of ±10° [6]. In addition, it was assumed that the pelvis would tilt forward because cup anteversion was larger in the group using the DAA [14, 15]. Therefore, a 10° increase was used as the reference. Explanatory variables were sex, age, BMI, Kellgren-Lawrence (KL) grade, Crowe’s classification, pelvic tilt before incision and preoperative ROM, flexion, abduction, adduction, external rotation and internal rotation, which surgeons assessed in the operating room.

### Statistical analysis

Changes in pelvic position were compared using a paired *t*-test. Univariate analyses of groups were estimated using the Fisher’s exact test or the Mann-Whitney U-test, since continuous variables were not normally distributed. Multivariate logistic regression analyses were performed to estimate risk of increased pelvic tilt and axial rotation. All variables in which *P* < 0.1 in the univariate analysis were considered independent variables. *P*-values less than 0.05 were considered statistically significant. All statistical analyses were performed using SPSS Ver. 26 (IBM Corp. Armonk, NY, USA) or EZR (Saitama Medical Center, Jichi Medical University, Saitama, Japan).

## Results

The study included 164 women and 36 men. The average age of study participants was 69.0 ± 9.6 years (range, 40–90 years). The average height of participants was 153.0 ± 8.9 cm (range, 134–180 cm), the average weight was 56.2 ± 12.0 kg (range, 35.8–94.6 kg), and the mean BMI was 24.1 ± 3.9 kg/m^2^ (range, 17.3–37.7 kg/m^2^). Based on Crowe’s classification, 193 hips were grade I, 6 were grade II, 1 was grade III, and no hips were grade IV. Based on the KL grading scale, 4 hips were grade 2, 45 were grade 3, and 151 were grade 4 (Table 1).

**Table 1:** Patient demographics

Sex (female/male)	164/36
Age (years)	69.0 ± 9.6 (40–90)
Height (cm)	153.0 ± 8.9 (134–180)
Weight (kg)	56.2 ± 12.0 (35.8–94.6)
BMI (kg/m^2^)	24.1 ± 3.9 (17.3–37.7)
K-L grade (1/2/3/4)	0/4/45/151
Crowe’s classification (I/II/III/IV)	193/6/1/0

Values are provided as means ± standard deviation (range from minimum to maximum)

*BMI* body mass index, *K-L* grade Kellgren-Lawrence grade

Intraoperative pelvic tilt increased by 7.6° ± 3.8° (95% CI, 7.1–8.2; range, − 5.0–19.0), while axial rotation increased by 3.2° ± 2.7° (95% CI, 2.7–3.7; range, − 13.0-12.0) (Table 2). Univariate analyses revealed that in female patients, the preoperative range of abduction and internal rotation were significantly greater than those in male patients, and BMI was significantly lower in patients with a large increase in pelvic tilt than in patients with no pelvic tilt increase (Table 3). Pelvic tilt at the beginning of the surgery was significantly increased in patients with large increases in pelvic axial rotation angle (Table 4). Other variables assessed did not significantly differ. Logistic regression analyses revealed that, BMI (OR, 0.889; 95% CI, 0.809–0.977; *p* = 0.014) and range of internal rotation (OR, 1.310; 95% CI, 1.002–1.061; *p* = 0.038) were predictors of a large increase in pelvic tilt. Logistic regression analysis indicated that pelvic tilt at the start of surgery was not a predictor of a large increase in axial rotation (Table 5).

**Table 2:** Pelvic movements (changes in pelvic tilt and axial rotation) from the start of surgery to cup placement

	At start of surgery	At cup placement	Mean of difference	SD of difference	95% CI of difference	*P* value ^a^
	(Min, Max)	(Min, Max)	(Min, Max)			
Pelvic tilt (°)	−7.4 (− 39.0, 7.0)	0.2 (−33.0, 16.0)	7.6 (−5.0, 19.0)	3.8	7.1–8.2	< 0.001
Axial rotation (°)	0.8 (−10.0, 7.0)	4.0 (−9.0, 12.0)	3.2 (−13.0,12.0)	3.7	2.7–3.7	< 0.001

*SD* standard deviation, *CI* confidence interval

^a^ Mann-Whitney U test

**Table 3:** Univariate analysis between increasing group and non-increasing group in change of pelvic tilt

	Increasing group	Non-increasing group	*P* value
	(51 hips)	(149 hips)	
Sex (female/male)	48/3	116/33	0.010^a^
Age (years)	68.8 ± 8.7	67.6 ± 10.0	0.687^b^
BMI (kg/m^2^)	23.3 ± 4.1	25.1 ± 3.7	0.001^b^
K-L grade (1/2/3/4)	0/1/14/36	0/3/31/115	0.248^a^
Crowe’s classification (I/II/III/IV)	0/48/2/1	0/145/4/0	0.193^a^
Pelvic tilt at the start of surgery (°)	−6.5 ± 5.9	−7.8 ± 6.6	0.167^b^
ROM (°)			
Flexion	111.9 ± 14.8	108.4 ± 17.5	0.315^b^
Abduction	35.7 ± 15.7	31.0 ± 10.1	0.044^b^
Adduction	18.5 ± 6.7	17.1 ± 6.3	0.188^b^
External rotation	43.5 ± 10.2	41.3 ± 11.3	0.399^b^
Internal rotation	33.2 ± 13.8	25.6 ± 13.5	0.001^b^

*BMI* body mass index, *K-L grade* Kellgren-Lawrence grade, *ROM* range of motion

^a^ Fisher’s exact test. ^b^ Mann-Whitney U test

**Table 4:** Univariate analysis between increasing group and non-increasing group in change of axial rotation

	Increasing group	Non-increasing group	*P* value
	(11 hips)	(189 hips)	
Sex (female/male)	9/2	155/34	1.000^a^
Age (years)	68.0 ± 10.4	67.9 ± 9.7	0.942^b^
BMI (kg/m^2^)	23.0 ± 4.6	24.7 ± 3.8	0.139^b^
K-L grade (1/2/3/4)	11/0/0/0	182/6/1/0	0.788^a^
Crowe’s classification (I/II/III/IV)	0/0/3/8	0/4/42/143	1.000^a^
Pelvic tilt at the start of surgery (°)	−5.5 ± 11.7	−7.5 ± 6.0	0.021^b^
ROM (°)			
Flexion	105.9 ± 22.3	109.5 ± 16.6	0.806^b^
Abduction	34.1 ± 13.9	32.1 ± 11.8	0.289^b^
Adduction	19.1 ± 11.1	17.4 ± 6.1	0.654^b^
External rotation	39.5 ± 13.1	42.0 ± 10.9	0.579^b^
Internal rotation	27.3 ± 21.4	27.6 ± 13.5	0.771^b^

*BMI* body mass index, *K-L grade* Kellgren-Lawrence grade, *ROM* range of motion

^a^ Fisher’s exact test. ^b^ Mann-Whitney U test.,

**Table 5:** Logistic regression for pelvic movements (enlargement in pelvic tilt and axial rotation)

Objective variable	Explanatory variable	Odds ratio	95% CI for odds ratio	P value	Nagelkerke R^2^
Enlargement in pelvic tilt	(constant)	0.628		0.745	0.150
	female	2.635	0.734–9.460	0.137	
	BMI	0.889	0.809–0.977	0.014	
	abduction	1.015	0.982–1.050	0.365	
	internal rotation	1.031	1.002–1.061	0.038	
Enlargement in axial rotation	(constant)	0.082			0.015
	pelvic tilt at the start of surgery	1.054	0.952–1.167	0.311	

*CI* confidence interval, *BMI* body mass index

## Discussion

This study investigated intraoperative pelvic tilt during THA via the DAA when the procedure was performed using an accelerometer-based navigation system. Our results showed that the pelvis tilted forward in the sagittal plane and leaned toward the surgical side in the axial plane. Low BMI and an increased range of internal rotation could indicate a > 10° increase in pelvic tilt. In contrast, there factor significantly predicted axial rotation.

Several studies reported that intraoperative pelvic movement occurs in the supine position during THA and that movement is associated with patient BMI. In the supine position, the relationship between BMI and pelvic motion associated with various approaches differ. Brodt et al. demonstrated that when the lateral approach was used, the pelvis tilted − 4.4° in the axial plane intraoperatively. Patients with a high BMI leaned more toward the opposite side than patients with lower BMI values. In contrast, there was no significant change observed in the sagittal plane [19]. Kamenaga et al. demonstrated that when the anterolateral supine approach (ALS) was used, the average forward change in pelvic tilt measured using HipAlign was 2.7°, and the average tilt toward the surgical side in axial rotation was 1.2°. The change in absolute axial rotation was significantly negatively correlated with BMI. The change in sagittal pelvic tilt was not associated with BMI [20].

To the best of our knowledge, this is the first study to review the association between pelvic movement and BMI when the DAA is applied. It is also the first to evaluate associations between clinical factors such as stage of osteoarthritis, ROM, and outcomes of the various surgical approaches in the supine position. Changes in axial rotation were the opposite when the anterior approach, including DAA and ALS, were used versus the lateral approach. Brodt et al. reported that in the lateral approach a strong upward traction was required to provide the surgeon with sufficient visualization [19]. In contrast, the anterior approach may have been strongly influenced by posterior strength retractors in patients with low BMI values. In this study, a low BMI was a predictor of increased pelvic tilt. The anterior distal retractor pulled strongly in order to not avoid interfering with soft tissues throughout cup implantation. At that time, the retractor was pressed downward from the front of the thigh (Fig. 1). As a result, it was inferred that a force was applied to the distal pelvis and the pelvis tilted forward. A Finite Element Method study of effects of mattresses indicated that when lying on a mattress in supine position, a thin person will sink deeper into mattresses than an obese person when the same force is applied [22]. Patients with a low BMI values are expected to move more on the surgical table when surgical procedures are performed, and the pelvis should tilt in a more anterior direction. Large internal rotation was a predictor of increasing pelvic tilt. Spinal flexibility in the sagittal plane was positively correlated with internal rotation ROM [23]. Large tilt angles tended to result in varied pelvic positioning. The ischial femoral ligament provided internal rotational stability [24], and external rotation that antagonized internal rotation was performed by external rotator muscles. The posterior capsule and short rotator were preserved in THA via DAA using a procedure that was based on a previous study [12]. In hip joints with small ROM of internal rotation, contracture of the posterior soft tissue may have suppressed the dorsal shift of the acetabula relative to the position of the femur. In cases where the range of internal rotation was large, the shift may not have suppressed movement, and the change of pelvic tilt tended to be large. BMI was not a predictor of unexpectedly increased axial rotation. In ALS, which is an anterior approach similar to DAA, a correlation between BMI and the absolute value of axial rotation was observed by Kamenaga et al. [20]. They described that the effect of traction may be large in patients with low BMI values. However, they reported that there was no correlation between real axial rotation values and BMI. The forces of the anterior and posterior retractors may offset each other, and one may not consistently dominate the other. Further research evaluating body pressure distribution and cadaver studies that measure the position of the femur and pelvis will be needed to elucidate mechanical mechanisms of the phenomenon.

Movement of the pelvis affects cup placement [16]. It has been reported that the DAA is associated a larger cup anteversion compared with the posterior approach [14, 15]. Forward tilting of the pelvis and deviation to the surgical side could cause increased anteversion because insertion of the cup against the surgical table may widen the target angle to a degree larger than that of the functional pelvic plane. When pelvic tilt increased by 7.6° and the cup was inserted using a mechanical guide with a radiographic inclination of 40° and anteversion of 15°, anteversion of the placed cup was approximately 5.7° larger than the target angle (calculated using Murry’s definition) [22]. If deviation in the axial plane increased, anteversion was further increased. The angle deviated significantly from the narrow safe zone that has been proposed in recent years, which specifies a range of ±5° [7, 8]. High cup anteversion caused anterior dislocation [9], which lead to concerns of anterior instability of the hip joint. To position the cup accurately, a device that tracked pelvic movements was useful [21]. However, many THAs are not computer-assisted [18].

This study had several limitations. First, it was a single-center study, and its results may not be generalizable. Results may have varied if the assistant used a retractor. However, in this study, retractors were fixed with retractor holding devices and weights. Second, ROM was manually measured under anesthesia, and did not truly represent the angle formed by the pelvis and the femur. The angle measured under anesthesia could have been exaggerated due to movement of the pelvis during measurement. Furthermore, low BMI values may have influenced degree of error associated with ROM measurement. Internal rotation angle remains a predictor of pelvic movement. Third, pelvic tilt with all retractors removed was not measured. It was also not confirmed whether pelvic movement and cup malposition were suppressed by removing all retractors and installing a cup. In addition, the threshold of a large increase of 10° might not have been appropriate. However, as the safe zone is controversial, clinically meaningful pelvic movement has not been determined. When anteversion based on an operative definition increased by 10°, anteversion based on a radiographic definition increased by 7.5°. We believe this discrepancy is a matter of concern. Finally, this cohort had low BMI values. The subjects in this cohort were all Japanese. In Japan, many individuals have a low BMI values. A cohort that has many obese patients may have experienced different outcomes. Future studies should aim to verify that patients with low BMI values and a large range of internal rotation have large cup anteversion values when a navigation system is not used.

## Conclusion

This study characterized pelvic forward tilt and axial rotation during THA via the DAA. Predictors of pelvic forward tilt were low BMI and high internal rotation range. The surgeon should pay attention to pelvic tilt changes when inserting the cup when no device, such as a navigation system, is used to track pelvic movements. This study has the potential to help surgeons identify patients at increased risk of intraoperative pelvic tilt change.

## Data Availability

The datasets used and/or analyzed during the current study are available from the corresponding author on reasonable request.

## Abbreviations

THA: Total hip arthroplasty
BMI: Body mass index
DAA: Direct anterior approach
ROM: Range of motion
ALS: Anterolateral supine approach
ASIS: anterior superior iliac spine
KL: Kellgren Lawrence
